# uPA and PAI-1 as biomarkers in breast cancer: validated for clinical use in level-of-evidence-1 studies

**DOI:** 10.1186/s13058-014-0428-4

**Published:** 2014-08-22

**Authors:** Michael J Duffy, Patricia M McGowan, Nadia Harbeck, Christoph Thomssen, Manfred Schmitt

**Affiliations:** UCD School of Medicine and Medical Science, Conway Institute, University College Dublin, Belfield, Dublin 4 Ireland; UCD Clinical Research Centre, St Vincent’s University Hospital, Elm Park, Dublin 4 Ireland; Breast Center, Department of Gynecology & Obstetrics, Klinikum Großhadern, Ludwig-Maximilians University, Marchioninistrasse 15, 81337 Munich, Germany; Department of Gynecology, Universitaetsklinikum Halle, Ernst-Grube-Strasse 40, 06097 Halle (Saale), Germany; Clinical Research Unit, Dept. Obstetrics @ Gynecology, Klinikum rechts der Isar, Technische Universitaet Muenchen, Ismaninger Strasse 22, 81675 Munich, Germany

## Abstract

Urokinase plasminogen activator (uPA) is an extracellular matrix-degrading protease involved in cancer invasion and metastasis, interacting with plasminogen activator inhibitor-1 (PAI-1), which was originally identified as a blood-derived endogenous fast-acting inhibitor of uPA. At concentrations found in tumor tissue, however, both PAI-1 and uPA promote tumor progression and metastasis. Consistent with the causative role of uPA and PAI-1 in cancer dissemination, several retrospective and prospective studies have shown that elevated levels of uPA and PAI-1 in breast tumor tissue are statistically independent and potent predictors of poor patient outcome, including adverse outcome in the subset of breast cancer patients with lymph node-negative disease. In addition to being prognostic, high levels of uPA and PAI-1 have been shown to predict benefit from adjuvant chemotherapy in patients with early breast cancer. The unique clinical utility of uPA/PAI-1 as prognostic biomarkers in lymph node-negative breast cancer has been confirmed in two independent level-of-evidence-1 studies (that is, in a randomized prospective clinical trial in which the biomarker evaluation was the primary purpose of the trial and in a pooled analysis of individual data from retrospective and prospective studies). Thus, uPA and PAI-1 are among the best validated prognostic biomarkers currently available for lymph node-negative breast cancer, their main utility being the identification of lymph node-negative patients who have HER-2-negative tumors and who can be safely spared the toxicity and costs of adjuvant chemotherapy. Recently, a phase II clinical trial using the low-molecular-weight uPA inhibitor WX-671 reported activity in metastatic breast cancer.

## Introduction

The ideal cancer biomarker should possess all or most of the following properties [1,2]:

- have an analytically validated assay for its measurement,
- have undergone validation for addressing a specific clinical problem,
- have been shown to have clinical utility such as improving patient outcome, enhancing quality of life, or reducing cost of care,
- have a cost-effective assay, and
- be a target for therapy.

In breast cancer, the biomarkers that best meet these criteria are the estrogen receptor (ER) [3] and the oncoprotein HER-2 (human epidermal growth factor receptor 2) [4]. Though not that widely used in the clinic at present, two other biomarkers - the serine protease urokinase plasminogen activator (uPA) and its inhibitor PAI-1 (plasminogen activator inhibitor-1) - also meet most of the above criteria. Indeed, uPA and PAI-1 are among the best validated biomarkers currently available for breast cancer, having undergone clinical validation and been shown to have clinical utility in two independent level-of-evidence-1 (LOE-1) studies [5,6]. These LOE-1 studies involved validation in both a multicenter randomized prospective clinical trial in which validation of the biomarkers was the primary aim of the trial and a pooled analysis of individualized data from both unpublished and published studies [7-9]. Promising data from animal models suggest that uPA, in addition to its biomarker role, may be a novel therapeutic target for the treatment of cancer [10-12]. The aims of this article are to provide an updated overview on uPA and PAI-1 as prognostic or predictive biomarkers in breast cancer (or both) and to discuss the potential therapeutic value of uPA in breast cancer.

## Biology of urokinase plasminogen activator and plasminogen activator inhibitor-1

Though referred to as a kinase, uPA does not possess any kinase activity. Rather, uPA is a protease, belonging to the serine peptidase family S1 of Clan PA, MEROPS identification S01.231, located on chromosome 10q24 [13,14]. Unlike most serine proteases and indeed most mammalian proteases, uPA has two notable characteristics. Firstly, in contrast to many proteases, uPA appears to have a restricted substrate specificity, its only identified biological substrate being the proenzyme plasminogen, which it converts to the enzymatically active serine protease plasmin. However, *in vitro* evidence suggests that uPA can cleave proteins other than plasminogen, such as fibronectin, alpha6 integrin, hepatocyte growth factor (HGF), urokinase plasminogen activator receptor (uPAR), and uPA itself [10,13,15]. In contrast to uPA, plasmin is a broad-spectrum protease, with the potential to cleave multiple substrates. In particular, it can degrade or remodel several extracellular matrix (ECM) components such as laminin, fibronectin, tenascin C, and osteopontin [16,17]. By cleaving ECM proteins, plasmin can release and thus activate growth factors sequestered at this site. Growth factors shown to be released from the ECM by plasmin include fibroblast growth factor 2, transforming growth factor-beta, and HGF [16]. These released and activated growth factors, following binding to their cognate receptors, can result in increased proliferation, migration, invasion, and metastasis.

In addition to its ability to cleave ECM substrates, plasmin can activate the zymogen forms of specific matrix metalloproteases (for example, MMP1, MMP2, MMP3, MMP9, MMP12, and MMP13) and the precursor form of uPA, pro-uPA [16]. These activated MMPs then can degrade the diverse forms of collagens, kallikrein-related peptidases, and other proteins in the ECM [18]. Thus, the uPA-mediated conversion of plasminogen to plasmin creates a powerful proteolytic system capable of remodeling the ECM and activating growth factors.

The second property of uPA that differentiates it from most other proteases is that it functions while bound with high affinity to a cell membrane receptor, known as the uPA receptor or uPAR (also known as CD87) [19]. The structure of uPAR is dissimilar from that of type I growth factor membrane receptors in lacking a transmembrane domain. Rather, uPAR is attached to the cell membrane via a glycosylphosphatidylinositol link. As uPAR lacks a transmembrane domain, it is unable to directly initiate downstream signaling. In order for such signaling to occur, uPAR must interact with other molecules such as epidermal growth factor receptor [20], platelet-derived growth factor receptor [21], specific integrins [22], or low-density lipoprotein receptor-related (LDLR) proteins [23]. Signaling pathways activated following uPA binding to uPAR include those involving the MAPK, Jak-Stat, and focal adhesion kinase systems [24]. These signaling systems regulate cell proliferation, migration, and metastasis. uPA thus may trigger cell signaling by two distinct mechanisms (that is, directly by binding to its receptor uPAR and indirectly by activation of plasmin which releases growth factors sequestered in the ECM) (see above).

To restrain its proteolytic function, uPA catalytic activity can be inhibited by two major endogenous inhibitors: PAI-1 and PAI-2. Both PAI-1 and PAI-2 belong to the serpin superfamily of protease inhibitors, PAI-1 being designated serpinE1 and PAI-2 as serpinB2. Of these two inhibitors, PAI-1 is the more rapidly acting, being 10- to 100-fold faster than PAI-2, at least *in vitro* [25]. Following binding of PAI-1 to the uPA-uPAR complex, the trimolecular complex undergoes endocytosis [26]. Endocytosis requires interaction with members of the LDLR family of the endocytosis receptors, such as LRP, LRP2, and very-low-density-lipoprotein receptor. After endoctytosis, the complex is degraded, followed by partial recycling of the free form of uPAR to the cell membrane [27].

## Role of urokinase plasminogen activator and plasminogen activator inhibitor-1 in cancer

Several studies using a variety of animal models have shown that uPA is causally involved in promoting cancer invasion and metastasis (for reviews, see [10,11]). Thus, early reports showed that the administration of antibodies to uPA, synthetic low-molecular-weight serine protease inhibitors, or small interfering RNAs against uPA decreased cancer progression [10,11]. Further confirmation of a role for uPA in metastasis was obtained with uPA or plasminogen-deficient mice. Thus, Bugge and colleagues [28] reported that a deficiency of plasminogen in the mouse mammary tumor virus-Pym breast cancer model reduced spontaneous metastasis without affecting tumor growth. Using the same animal model, Almholt and colleagues [29] found that a deficiency in uPA resulted in the reduced formation of lung and lymph node metastasis. As in the report by Bugge and colleagues, tumor growth was not affected in this study.

As metastasis is a multistep event, it was important to identify the specific step or steps in which uPA was involved. Using the chick embryo system, Ossowski [30] showed that uPA was involved in an early step in the metastasis of Hep3 tumor cells. Similarly, using prostate cancer cells, Bekes and colleagues [31] showed that uPA participates at an early phase of cancer dissemination (that is, in the initial escape of tumor cells from the primary site). This escape of tumor cells was found to be dependent on uPA-mediated plasmin activation and degradation of the ECM protein fibronectin. In this model, prevention of tumor escape was blocked by inhibition of pro-uPA activation.

It might be expected that, based on its ability to inhibit uPA activity, PAI-1 would suppress cancer progression. However, consistent data from clinical studies (see below) suggest that PAI-1 at levels found in tumor extracts is involved in mediating cancer progression. Indeed, direct evidence of a role for PAI-1 in cancer was recently obtained when Masuda and colleagues [32] reported that a specific inhibitor of PAI-1 blocked angiogenesis and tumor progression in an animal model. The PAI-1 inhibitor appeared to mediate its anti-tumor effects by interacting with host PAI-1.

A possible mechanism by which PAI-1 promotes cancer progression is by enhancing angiogenesis. Evidence of a role for PAI-1 in new blood vessel formation first emerged when it was shown that PAI-1 deficiency in mice resulted in defective angiogenesis [33-35]. In one of these reports, PAI-1 was found to act by stimulating migration of endothelial cells from perivascular areas rich in the ECM protein vitronectin to sites rich in fibronectin [35]. The mechanism by which PAI-1 promotes angiogenesis may relate to its ability to protect the ECM from excessive degradation, as this structure provides a scaffold for endothelial cell migration and formation of capillaries [34].

A further mechanism by which PAI-1 may enhance cancer progression is by blocking apoptosis and thus enhancing cell survival. PAI-1 was first reported to be anti-apoptotic when Kwaan and colleagues [36] found that the addition of recombinant PAI-1 to tumor cells *in vitro* inhibited cytotoxic drug-induced apoptosis. Subsequently, PAI-1 was shown to protect endothelial cells [37] as well as several different types of tumor cells from apoptosis. This blockage of apoptosis was found to be dependent on uPA-mediated activation of plasmin and the interaction of FasL with Fas [37].

## Urokinase plasminogen activator and plasminogen activator inhibitor-1 as biomarkers in breast cancer

### Analytical validation of urokinase plasminogen activator and plasminogen activator inhibitor-1 assays

As mentioned at the start of this article, an essential requirement for clinical use of a biomarker is analytical validation of the assay to be used for its measurement. Analytical validation ensures that an assay is reproducible and stable and has adequate sensitivity for detecting the biomarker of interest in the fluid to be used for its measurement [38,39]. In addition, to ensure reproducibility between different laboratories, it is important that clinically used assays be evaluated in external quality assessment (EQA) programs [38].

Several different methodologies, including enzyme-linked immunosorbent assay (ELISA) and immunohistochemistry at the protein level and RT-PCR at the mRNA level, have been used to measure uPA and PAI-1 in research laboratories. Of these methodologies, the only method subjected to analytical validation is ELISA [40,41]. In an early analytical study, Benraad and colleagues [40] evaluated six different ELISAs for uPA measurement. Although these different assays were developed independently and used different antibodies and standards, good correlations were found between the different systems. The absolute levels of uPA detected, however, varied between the different assays. Importantly, all methods gave acceptable within-assay precision [40,41]. Thus, using quality control samples, the between-assay coefficient of variation (CV) varied between 5.0% and 9.8% for uPA and between 5.4% and 5.8% for PAI-1 [41]. Furthermore, all of the assays were adequately sensitive to detect uPA levels in extracts of breast cancer [40]. One of these assays - Femtelle uPA/PAI-1 (Sekisui Diagnostics LLC, formerly American Diagnostic Inc., Lexington, MA, USA) - was later evaluated in an EQA program. In this multicenter study, which involved six laboratories in Germany, the between-laboratory CV varied between 6.2% and 8.2% for uPA and between 13.2% and 16.6% for PAI-1 [41]. More recently, in a randomized trial, CVs of 12% in an EQA program were reported for both uPA and PAI-1 [42]. These CVs obtained with manual ELISAs would be regarded as acceptable for clinical use.

### Clinical validation

Clinical validation ensures that a positive biomarker test result is associated with a particular disease or clinical endpoint [43]. Appropriate endpoints for relating uPA and PAI-1 include disease-free interval, overall survival, or response to therapy. Given their involvement in cancer progression, uPA and PAI-1 were logical candidates for evaluation as potential prognostic biomarkers in patients with cancer [44]. In a preliminary finding, Duffy and colleagues [45] first reported that breast cancer patients with high tumor levels of uPA catalytic activity had a significantly shorter disease-free interval than patients with low activity levels.

These preliminary findings were soon confirmed when Jänicke and colleagues [46,47], using an immunoassay, reported that high uPA levels were associated with adverse outcome in patients with breast cancer. Jänicke and colleagues [48] later found that, in addition to uPA, elevated levels of PAI-1 predicted poor outcome as well. Subsequently, more than 20 independent groups confirmed these initial findings [49-80]. These studies also showed that uPA and PAI-1 were statistically independent prognostic biomarkers for patients with breast cancer and importantly were prognostic in the subset of patients with lymph node-negative disease [47,52,56,60,61,63,67,68]. The consistency of these findings across multiple patient populations clearly demonstrates that both uPA and PAI-1 are related to outcome in women with breast cancer, including the subgroup with lymph node-negative disease.

In addition to having a prognostic impact in breast cancer, uPA and PAI-1 measurements in breast cancer appear to possess therapy predictive value, especially in predicting benefit from cyclophosphamide-methotrexate-5-fluorouracil (CMF) in the adjuvant setting. In an early prospective study (n = 761), Harbeck and colleagues [72] reported that, although uPA and PAI-1 were associated with outcome in patients who did not receive systematic adjuvant therapy, this prognostic effect was lost in patients who received adjuvant chemotherapy. Further evidence of a chemotherapy predictive potential for uPA/PAI-1 was obtained by using data from two separate sites (n = 3,424), which showed that breast cancer patients with high levels of the biomarkers derived an enhanced benefit from adjuvant chemotherapy (mostly CMF) than those with low levels [79]. In addition to showing a benefit from adjuvant CMF, high levels of uPA and PAI-1 have been associated with response to adjuvant anthracycline-based therapy [81]. Further evidence of a predictive impact for uPA and PAI-1 was the finding that detection of the uPA/PAI-1 complex was also associated with benefit from adjuvant chemotherapy [80].

### Demonstration of clinical utility

Although the above findings, when taken together, provide strong evidence that uPA and PAI-1 were independent prognostic biomarkers in breast cancer, they were insufficient for these biomarkers to be recommended for routine clinical use. In order for emerging biomarkers to progress to the clinic, it is now widely accepted that, in addition to analytical and clinical validation, demonstration of clinical utility in a level-of-evidence (LOE-1) study is necessary. LOE-1 studies include validation in a randomized prospective trial in which the biomarker evaluation rather than an investigational drug is the primary purpose of the trial, retrospective employment of archival specimens from a previously conducted prospective trial, or a meta-analysis/pooled analysis of retrospective and prospective studies [5,6].

Uniquely for cancer prognostic biomarkers, uPA and PAI-1 have undergone validation in two separate LOE-1 studies; that is, both have been validated in a prospective randomized controlled trial (NCT1317108) and in a pooled analysis of individualized patient characteristics. Validation in the prospective randomized trial involved multiple centers in Germany [7,8]. In this trial (dubbed the Chemo-N0 trial), uPA and PAI-1 were measured by ELISA (Femtelle) in 556 patients with newly diagnosed axillary node-negative breast cancer. To ensure accuracy and precision for the uPA and PAI-1 assays, participation in EQA for all participating centers was mandatory.

Pre-validated optimized cutoff points were used, and patients with low concentrations of uPA (less than 3 ng/mg of protein) and PAI-1 (less than 14 ng/mg of protein) were subjected to surveillance without receiving adjuvant systemic therapy. On the other hand, women with high concentrations of uPA (at least 3 ng/mg of protein) or PAI-1 (at least 14 ng/mg of protein) or both were randomly allocated to adjuvant chemotherapy (CMF) or to surveillance without systemic therapy. Patients refusing to be randomly assigned underwent observation.

At the first interim analysis after a median follow-up period of 32 months, patients with low concentrations of uPA and PAI-1 had a significantly lower 3-year recurrence rate (that is, 6.7%) than those with high concentrations of uPA or PAI-1 or both (that is, 14.7%) (*P* = 0.006) [7]. These interim findings were recently confirmed following the 10-year analysis of this trial (median follow-up of 113 months) [8]. With this extended follow-up period, the disease recurrence rates in the absence of any adjuvant therapy (chemotherapy or endocrine therapy) were 12.9% for patients with low concentrations of uPA/PAI-1 and 23% for women in the high-uPA/PAI-1 group (*P* = 0.011). Considering these findings, the authors concluded that measurement of uPA and PAI-1 could identify almost half of the patients with lymph node-negative breast cancer as being at low risk for recurrence and thus being able to avoid the toxicity and costs of adjuvant chemotherapy.

The second LOE-1 study to have validated the prognostic utility of uPA and PAI-1 in breast cancer involved a pooled analysis of individual patient demographics data from 18 European datasets (n = 8,377) and was performed by the European Organization for Research and Treatment of Cancer (EORTC) Receptor and Biomarker group [9]. All centers used immunoassay to measure uPA and PAI-1 and were involved in ongoing quality assurance programs for these biomarkers. Baseline established clinical and histological factors in the multivariate analysis included tumor stage, tumor grade, number of lymph nodes involved, hormone receptor status, and patient age. Rather than using data exclusively from published studies which may result in the introduction of bias (as positive studies are more likely to be accepted for publication than negative findings), the study included both published (n = 11) and unpublished (n = 7) studies in the pooled analysis. Pooling of the data followed by multivariate analysis showed that for both lymph node-positive and lymph node-negative patients, increased concentrations of uPA and PAI-1 were independently associated with poor outcome. Importantly, both biomarkers were also prognostic in untreated (that is, without systemic therapy) lymph node-negative patients, indicating that these proteins were pure prognostic factors for this subgroup of patients. It should be stated that this is one of the very few studies to have investigated prognostic biomarkers in patients who did not receive adjuvant systemic treatment.

As with their prognostic impact, the therapy predictive value of uPA/PAI-1 has been confirmed in the above two LOE-1 studies. Thus, in the first interim analysis of the Chemo-N0 trial, high-risk lymph node-negative patients who had elevated concentrations of uPA or PAI-1 (or both) and who received chemotherapy exhibited a significantly lower probability of disease recurrence than those who had high concentration and who were subjected to surveillance alone (for the per-protocol analysis, relative risk (RR) = 0.27, *P* = 0.016; for the intention-to-treat analysis, RR = 0.56, *P* = not significant) [7]. This lack of significance in the intention-to-treat group may have resulted because some patients did not adhere to the trial protocol.

Confirmation of the above finding was obtained in the 10-year follow-up analysis [8]. With this longer follow-up period, high-risk patients randomly assigned to chemotherapy also exhibited a significantly lower probability of disease recurrence than those randomly assigned for observation only (in the per-protocol analysis, hazard ratio (HR) = 0.48, *P* = 0.019; in the intention-to-treat analysis, HR = 0.74, *P* = not significant). Confirmation of the chemotherapy predictive role for uPA and PAI-1 was found by using the pooled analysis of 18 datasets, referred to above [82].

## Measurement of urokinase plasminogen activator and plasminogen activator inhibitor-1 are cost-effective tests

Increasingly, in recent years, many governments and insurance companies are requiring economic analyses of the cost-effectiveness of new therapies and new tests prior to approval for clinical use. An economic analysis thus is becoming an additional hurdle (that is, in addition to analytical validation, clinical validation, and demonstration of clinical utility) before measurement of a new biomarker can be approved for funding. From the above data, it is clear that an upfront knowledge of the uPA/PAI-1 levels has the potential to reduce the use of unnecessary adjuvant chemotherapy in a subset of patients with lymph node-negative breast cancer.

Direct evidence that uPA/PAI-1 measurement is cost-effective and indeed cost-saving was recently shown in a prospective multicenter study involving 93 lymph node-negative and ER-positive breast cancer patients [83]. In this economic analysis, measurement of uPA/PAI-1 was found to decrease the use of adjuvant chemotherapy in 35 (37.6%) of the 93 patients investigated. Overall, measurement of the two biomarkers led to a total cost saving of €255,534. Considering the biomarker measurement cost of €288 per sample, the authors calculated that uPA/PAI-1 testing was cost-effective with a return on investment ratio of 8.4:1 [83].

### Current status of urokinase plasminogen activator and plasminogen activator inhibitor as prognostic and predictive biomarkers

To the best of our knowledge, uPA and PAI-1 are the first breast cancer biomarkers to have their prognostic and predictive utility validated in either a randomized prospective trial or a pooled analysis of individualized data from both published and unpublished data (that is, in two independent LOE-1 studies) [5,6]. In recent years, several multigene profiles have been proposed for determining prognosis in breast cancer [84] and indeed some of these - for example, Oncotype Dx (Genomic Health, Redwood City, CA, USA) and MammaPrint (Agendia, Irvine, CA, USA) - are currently in clinical use. However, at this stage, none of the gene signatures has been validated for clinical utility in LOE-1 studies, although such trials are ongoing for Oncotype Dx (NCT00310180 and NCT01272037) and MammaPrint (NCT00433589). It is of interest that preliminary results from a subset of patients (n = 314) participating in the WSG Plan B trial showed good agreement between uPA/PAI-1 and Oncotype Dx in high-risk patients but less agreement in those at low or intermediate risk of recurrence [85].

Because of their comprehensive validation, measurement of uPA and PAI-1 in breast cancer is now recommended by several expert panels in the US and Europe. Expert panels recommending clinical use of the assays include the American Society of Clinical Oncology [86], the National Academy of Clinical Biochemistry (US) [87], the European Group on Tumor Markers [88], the European Society of Medical Oncology [89], and the German Gynecological Oncology Group/Arbeitsgemeinschaft Gynäkologische Onkologie [90] (Table 1).

**Table 1:** **Expert panels that include uPA and PAI-1 measurements in their guidelines**

**Panel**	**Recommendation/Statement**	**Reference**
ASCO	uPA/PAI-1 measured by ELISAs may be used for the determination of prognosis in patients with newly diagnosed, node-negative breast cancer. Low levels of both markers are associated with a sufficiently low risk of disease recurrence, especially in steroid hormone receptor-positive women who will receive adjuvant endocrine therapy and who will receive only minimal additional benefit from chemotherapy. Furthermore, CMF-based adjuvant chemotherapy, compared with observation alone, provides substantial benefit in patients with a high risk of disease recurrence as determined by high levels of uPA and PAI-1.	[86]
NACB	Testing for uPA and PAI-1 may be carried out to identify lymph node-negative patients who do not need or are unlikely to benefit from adjuvant chemotherapy. Measurement of both proteins should be performed because the information provided by the combination is superior to that from either alone. Lymph node-negative patients with low levels of both uPA and PAI-1 have a low risk of disease recurrence and thus may be spared from the toxic side effects and costs of adjuvant chemotherapy. Lymph node-negative women with high levels of either uPA or PAI-1 should be treated with adjuvant chemotherapy.	[87]
EGTM	Recommends uPA and PAI-1 for determining prognosis in breast cancer, especially in the group of patients with lymph node-negative disease. Validated ELISAs (that is, validated for both analytical and clinical performance) should be used for determining these proteins.	[88]
ESMO	uPA-PAI-1, a marker of tumor invasiveness, has been validated in prospective clinical trials as a prognostic marker for both node-negative and node-positive breast cancer and can be used in treatment decision making for early breast cancer.	[89]
AGO	Recommends uPA and PAI-1 for determining prognosis in breast cancer, especially in the group of patients with lymph node-negative disease. It also acknowledges the predictive impact of the test.	[90]

AGO, Arbeitsgemeinschaft Gynäkologische Onkologie (German Gynecological Society); ASCO, American Society of Clinical Oncology; CMF, cyclophosphamide-methotrexate-5-fluorouracil; EGTM, European Group on Tumor Markers; ELISA, enzyme-linked immunosorbent assay; ESMO, European Society of Clinical Oncology; NACB, National Academy of Clinical Biochemistry (US); PAI-1, plasminogen activator inhibitor-1; uPA, urokinase plasminogen activator.

Although the assays for uPA and PAI-1 have been technically and clinically validated, these biomarkers are still infrequently used for clinical purposes. One of the reasons for this is that the original assays used for detecting these proteins required relatively large amounts of tumor tissue, limiting their application to patients with small cancers. Recently, however, Thomssen and colleagues [91] reported a strong and significant relationship between uPA and PAI-1 protein concentration in needle biopsy tumor tissue and level in the corresponding larger tumor samples (for uPA, r = 0.789; for PAI-1, r = 0.907; *P* <001 for both). Results from the needle biopsy samples gave a positive predictive value of 0.94 and a negative predictive value of 1.00 when compared with findings from the corresponding larger specimens. This report shows the feasibility of using a commercially available ELISA to quantitate uPA and PAI-1 levels in needle biopsies of breast cancer tissue.

A further reason for the limited clinical use of uPA/PAI-1 is that their measurement requires fresh or freshly frozen tumor tissue. Currently, however, attempts are ongoing to develop immunohistochemistry assays on formalin-fixed and paraffin-embedded tissues for predicting patient outcome. Indeed, a recent report showed that uPA and PAI-1 levels as measured by immunohistochemistry on formalin-fixed and paraffin-embedded tissue correlated significantly with values from a validated ELISA [92]. However, it remains to be shown whether immunohistochemically determined uPA and PAI-1 levels on fixed tissue predict patient outcome as accurately as values measured with ELISA.

## Ongoing clinical breast cancer trials using urokinase plasminogen activator and plasminogen activator inhibitor-1

Currently, two randomized prospective trials are investigating the predictive utility of uPA/PAI-1 for newer forms of breast cancer systemic therapies. One of these, which is known as the NNBC-3 trial and which has enrolled 4,147 patients, is comparing fluorouracil (5-FU), epirubicin, and cyclophosphamide followed by docetaxel (3xFEC-3xDoc; FEC-D) with 5-FU, epirubicin, and cyclophosphamide (6xFE100C; FEC) as adjuvant chemotherapy for high-risk lymph node-negative patients (NCT01222052) [93,94]. In this trial, unlike in the Chemo-N0 trial mentioned above, steroid hormone receptor-positive patients may receive endocrine therapy. Risk of disease recurrence was determined by clinicopathological criteria [95] or by a combination of uPA/PAI-1 and clinicopathological criteria. In this trial, as in the Chemo-N0 trial, uPA and PAI-1 levels were measured by the Femtelle ELISA and all participating laboratories undertook EQA. The first results are expected soon.

Another randomized trial involving measurement of uPA and PAI-1 (WSG Plan B trial) has completed recruitment with 2,448 high-risk node-negative and node-positive patients [42,96]. The aim of this trial is to compare an anthracycline- and taxane-based adjuvant chemotherapy combination with an anthracycline-free taxane-based regimen in patients with HER-2-negative breast cancer (NCT01049425). As part of this trial, the prognostic and predictive potential of uPA/PAI-1 will be compared with that of Oncotype DX.

## Urokinase plasminogen activator as a target for anticancer treatment

In addition to appropriate validation and demonstration of clinical utility, a desirable property of a cancer biomarker molecule is its capacity to function as a therapeutic target. Extensive data from animal models suggest that uPA may indeed be a target for the treatment of cancer [10,11,15]. Two main approaches have been used to block uPA: inhibition of its catalytic activity by selective low-molecular-weight inhibitors and prevention of uPA from binding to uPAR by using antagonistic peptides or antibodies (for detailed reviews, see [10,11,15]). Although both of these approaches show promising efficacy in animal models [10,11,15], low-molecular-weight catalytic inhibitors have been the more investigated approach in clinical trials.

Two low-molecular-weight synthetic inhibitors targeting serine proteases have undergone evaluation in clinical trials: WX-UK1 and WX-671 (also known as Mesupron or upamostat) (Wilex, München, Germany). Whereas WX-UK1 has to be administered intravenously, upamostat, which is a pro-drug of WX-UK1, can be given orally. After administration, upamostat is rapidly metabolized to the active drug, WX-UK1. In phase I trials, these two inhibitors were found to be well tolerated, and no serious side effects were reported [11,97].

Since upamostat has the advantage that it can be administered orally, it has been further investigated in phase II clinical trials. In one of these trials involving 132 patients with first-line metastatic breast cancer, the combination of upamostat and capecitabine was compared with capecitabine alone (NCT00615940) [98]. Administration of upamostat and capecitabine resulted in median progression-free survival (PFS) values of 8.3 months (95% confidence interval (CI) 5.6 to 9.6) in the total study population and 7.5 months (95% CI 4.2 to 12.8) in the control group given only capecitabine. However, in the group of patients who had received prior adjuvant chemotherapy, PFS increased from 4.3 months (95% CI 2.6 to 9.7) in those treated with capecitabine alone to 8.3 months (95% CI 5.6 to 10.9) in the group receiving upamostat and capecitabine. In addition, overall response rate was higher in the group receiving the combination therapy compared with those receiving capecitabine alone (20% versus 12% at week 24). Importantly, the combination of upamostat and capecitabine was reported to be safe and well tolerated, with no unexpected toxicities other than those attributable to capecitabine.

Upamostat has also been investigated in a randomized phase II trial in patients with locally advanced pancreatic cancer [99]. In this trial, 93 patients were randomly assigned to receive gemcitabine either alone or in combination with a daily dose of 200 or 400 mg upamostat. Of the three groups, the combination of the higher dose of upamostat and gemcitabine appeared to be most efficacious with regard to improving patient survival. Overall, upamostat was again well tolerated, the most common side effects being asthenia, fever, and nausea. It is unfortunate that neither of the above trials included prior measurements of tumor uPA levels. Were tumor uPA levels measured upfront and only those patients with high levels treated, it is likely that response rates would be higher. We recommend that, where possible, further trials with anti-uPA treatments involve prior measurement of uPA protein expression levels.

## Conclusions

The data presented above, especially the results from the Chemo-N0 randomized prospective trial [7,9], clearly show that lymph node-negative breast cancer patients as identified with uPA/PAI-1 measurements have an excellent outcome, despite not receiving adjuvant chemotherapy. Indeed, after 10 years of follow-up, only 10% of these patients have died and 13% had developed a recurrence/metastasis [9]. As previously pointed out [9], had these patients received hormone therapy, their 10-year overall survival likely would have exceeded 90%. Combined with its ability to identify lymph node-negative breast cancer patients who can be spared adjuvant chemotherapy (that is, women with low levels of uPA/PAI-1), high concentration of these biomarkers can select women who are likely to benefit from such therapy. Clearly, therefore, measurement of uPA and PAI-1 can help toward personalizing treatment for women diagnosed with lymph node-negative breast cancer.

For clinicians managing breast cancer patients, the key question is which of the available multigene/multiprotein tests is best. At present, the answer to this question is unclear. In the absence of a clear answer, the following might be considered in clinical decision making. Of the available multi-parameter tests, the best validated is uPA/PAI-1 [7-9]. Indeed, a commercial assay for measuring both uPA and PAI-1 is now available (that is, Femtelle, American Diagnostic Inc./Sekisui Diagnostics). Furthermore, this assay has received the CE mark for use in Europe. The test is widely used in Germany and to a lesser extent in France. The main reason for the limited use is that the Femtelle assay requires fresh/freshly frozen tumor tissue. Other multi-parameter tests such as Oncotype DX and MammaPrint can be performed on formalin-fixed, paraffin-embedded tissue and thus can offer more convenient assays. Hopefully, in the future, the various multi-parameter tests can be compared for their prognostic and predictive benefits as well as their cost-effectiveness.

## Note

This article is part of a series on *Recent advances in breast cancer treatment*, edited by Jenny Chang. Other articles in this series can be found at http://breast-cancer-research.com/series/treatment.

## Abbreviations

5-FU: fluorouracil
CI: confidence interval
CMF: cyclophosphamide-methotrexate-5 fluorouracil
CV: coefficient of variation
ECM: extracellular matrix
ELISA: enzyme-linked immunosorbent assay
EQA: external quality assessment
ER: estrogen receptor
HER-2: human epidermal growth factor receptor 2
HGF: hepatocyte growth factor
HR: hazard ratio
LDLR: low-density lipoprotein receptor
LOE-1: level of evidence 1
LRP: low-density lipoprotein receptor-related protein
MMP: matrix metalloprotease
PAI-1: plasminogen activator inhibitor-1
PFS: progression-free survival
RR: relative risk
uPA: urokinase plasminogen activator
uPAR: urokinase plasminogen activator receptor
